# Antifatigue Effect of *Panax Notoginseng* Leaves Fermented With Microorganisms: *In-vitro* and *In-vivo* Evaluation

**DOI:** 10.3389/fnut.2022.824525

**Published:** 2022-02-22

**Authors:** Min Yang, Liang Tao, Cun-Chao Zhao, Zi-Lin Wang, Zhi-Jin Yu, Wen Zhou, Yan-Long Wen, Ling-Fei Li, Yang Tian, Jun Sheng

**Affiliations:** ^1^College of Food Science and Technology, Yunnan Agricultural University, Kunming, China; ^2^Engineering Research Center of Development and Utilization of Food and Drug Homologous Resources, Ministry of Education, Yunnan Agricultural University, Kunming, China; ^3^National Research and Development Professional Center for Moringa Processing Technology, Yunnan Agricultural University, Kunming, China; ^4^Key Laboratory of Pu-erh Tea Science, Ministry of Education, Yunnan Agricultural University, Kunming, China

**Keywords:** *Panax notoginseng* leaves, microbial, fermentation, weight-loading swimming, antifatigue, antioxidant

## Abstract

Fatigue is a common physiological phenomenon caused by many complicated factors. Excessive fatigue will lead to a series of uncomfortable reactions and damage body health. *Panax notoginseng* leaves (PNL) is a new resource food that good for soothing nerves, nourishing the heart, and strengthening the spleen. Microbial fermentation could increase the content of bio-ingredients and produce new active ingredients. However, the effect of fermented *P. notoginseng* leaves (FPNL) on antifatigue and the molecular mechanisms remain to be elucidated. Thus, in this study, we evaluated the antifatigue effect of co-fermented *P. notoginseng* leaves by *Saccharomyces cerevisiae* and *Bacillus subtilis in-vitro* and *in-vivo*, and its mechanism was further elucidated. The results showed that FPNL exhibited higher saponins, organic phenolic acids content, and antioxidant activity than PNL. FPNL improved ISO-induced H9c2 myocardial cell damage by alleviating apoptosis (modulating Bax and Bcl-2 protein expression) and reducing antioxidant activity *in-vitro*. Moreover, *in-vivo* experiment showed that FPNL significantly prolonged the weight-loading swimming time of mice. After gavaged FPNL, the levels of liver glycogen (LG) and serum lactate dehydrogenase (LDH) activity were increased in mice. In contrast, the levels of blood urea nitrogen (BUN), lactate acid, and malondialdehyde (MDA) were decreased. In summary, our results indicated that FPNL showed a good antifatigue effect *in-vivo* and *in-vitro*.

## Introduction

Fatigue refers to a physiological state in which the body cannot maintain a certain level of function or an organ cannot maintain a predetermined exercise intensity. This phenomenon is mainly caused by excessive energy consumption in the body and the accumulation of metabolic waste such as lactic malondialdehyde (1). Fatigue can be divided into secondary fatigue, physiological fatigue, or chronic fatigue. Secondary fatigue is due to sleep disorders, depression, overwork, or drug side effects. Physiological fatigue is caused by lack of rest, physical labor, or mental reasons.

Chronic fatigue refers to fatigue that persists for more than 6 months. All types of fatigue can lead to health damage (2). In addition, fatigue is also divided into central fatigue and peripheral fatigue. Central fatigue is triggered by factors related to the central nervous system, brain, spinal cord, and motor neurons, while peripheral fatigue is related to muscle weakness caused by changes in the neuromuscular junction or distal end (3). The accumulation of lactic acid (LD) and ammonia metabolites, blood sugar, and tissue glycogen consumption are the leading cause of peripheral fatigue (4). However, the continuous supply of energy resources and the elimination of metabolites could alleviate peripheral fatigue (5). Fatigue is becoming increasingly severe in modern society, leading to aging, cancer, depression, multiple sclerosis, Parkinson's syndrome, and other diseases. Research has shown that antifatigue drugs may cause adverse reactions or toxic effects (6). While regular exercise and a balanced diet are considered healthy methods to relieve fatigue. However, people are also looking for other antifatigue methods. Some studies have shown that herbal medicine has unique advantages in eliminating fatigue. Therefore, developing natural antifatigue products to improve exercise ability and relieve fatigue is a common concern in the future.

*Panax notoginseng* is a perennial herb that showed a good function at promoting blood circulation, relieving swelling, and protecting the human liver (7). The primary chemical constituents of *P. notoginseng* are saponins, which can be classified into four types depending on the glycine content: 20 (S)—protopanoxadiol types (PPD), 20 (S)—protopanaxatriol types (PPT), C17 side chain variation types and other kinds types. For a long time, *P. notoginseng* has been involved in a wide range of medical fields. With the continuous utilization of natural resources, *P. notoginseng* leaves (PNL) as a new food material have been gradually discovered and used by many studies. Studies have found that PNL is rich in various active ingredients, such as promoting blood circulation, eliminating stasis, relieving pain, reducing inflammation, and promoting digestion (8–10). In addition, PNL contains seven kinds of essential amino acids and is rich in vitamin C, nicotinamide, folic acid, biotin (8). Meanwhile, the saponin content of PNL was approximately 9%. These saponins had different biological activities (11). Therefore, the development and utilization of PNL have become a research hotspot.

Microbial fermentation could secret various biological enzymes to decompose the structure of plant cells (12), increase the content of active ingredients in plants (13). In this process, microorganisms metabolize macromolecules into small molecules for easy absorb. Therefore, microbial metabolism could improve the utilization rate of active ingredients (14). A lot of foods are fermented or enriched in probiotics to be evaluated as possible carriers of these beneficial microorganisms. Several species of *Lactobacillus* and *Bifidobacterium* have become the most commonly used probiotic strains in these food products. Other microorganisms such as *Saccharomyces cerevisiae, Enterococcus, Bacillus*, and *Escherichia* are also applied in functional food (15). It was shown that after fermenting by *Bacillus subtilis*, the ginsenoside Rh4 of *P. notoginseng* roots was obtained (16). *Panax notoginseng* leaves fermented by microorganisms were increased polysaccharide contents and significantly showed antiinflammatory effects (17). Moreover, PNL fermented by *Lactobacillus* significantly improved the anti-liver cancer activity (14). Therefore, fermentation is one of the most convenient techniques in biocatalytic processes. During fermentation, microbial metabolism can transform raw materials into products with specific health-promoting properties.

This study investigated the contents of total saponins, total polyphenols, and organic phenolic acids in PNL and fermented *P. notoginseng* leaves (FPNL). The stability of bioactive substances from FPNL has been studied with ABTS and 2,2-diphenyl-1-picrylhydrazyl (DPPH) analysis. Then a fatigue injury model of H9c2 cardiomyocytes was constructed with isoproterenol (ISO), and the protective effects of FPNL and PNL on H9c2 cells were investigated. Furthermore, the antifatigue effect of FPNL was studied by a weight-loading swimming experiment in mice. Finally, the swimming time and antifatigue-related biochemical indices were detected to evaluate the antifatigue of FPNL *in-vivo and in-vitro*.

## Materials and Methods

### Plant Materials and Reagents

*Panax notoginseng* leaves were collected from the organic *P. notoginseng* planting base at Yunnan Agricultural University (Lan Cang, Yunnan, China). *Lactobacillus acidophilus* (CICC 20710), *Lactobacillus reuteri* (CICC 6226), *Lactobacillus plantarum* (CICC 194165), *Pichia kluyveri* (CICC 32845), *S. cerevisiae* (CICC 31393), and *B. subtilis* (CICC 22459) were purchased from the China Center of Industrial Culture Collection; *S. cerevisiae* (GIM2.43) and *Rhizopus oryzae* (CICC 41441) were purchased from Guangdong Microbial Culture Collection Center. All strains were stored at −80°C; 3-4,5-dimethylthiazole-z-yl-3,5-diphenyltetrazolium bromide (MTT), trypsin, dimethyl sulfoxide (DMSO), and phosphate-buffered saline (PBS) were purchased from Solarbio Technology Co., Ltd. (Beijing, China) and stored at −20°C; DMEM and fetal bovine serum (FBS) were obtained from Invitrogen Technology (Gaithersburg, USA). BCA protein quantitative kit was acquired from Beyotime Biotechnology Co., Ltd. (Shanghai, China). Antibodies against Bax and Bcl-2 were acquired from Cell Signaling Technology (Beverly, USA); rabbit and mouse antibodies were acquired from Abcam (NY, USA). A liver glycogen (LG) assay kit (A043-1-1), LD assay kit (A019-2-1), lactate dehydrogenase (LDH) assay kit (A020-2-2), malondialdehyde (MDA) assay kit (A003-1-2), and urea assay (BUN) kit (C013-2-1) were purchased from Nanjing Jiancheng Bioengineering Institute (Jiangsu, China).

### Preparation of FPNL

The strains were cultured in MRS medium for 24 h, then sub-cultured all strains three times. Strains of *Lactobacillus* spp. were cultured under anaerobic conditions in a Bugbox anaerobic chamber (Ruskin Technology, USA). The PNL were dried in an oven, crushed, and screened for 40 mesh. *Panax notoginseng* leaves was extracted by microwave-assisted method (18). The PNL powder (10 g) was immersed in 200 ml UP water, ultrasonicated (200 W) at 110°C for 15 min, then 1% glucose was added, sterilized at 121°C for 20 min, and cooled for reserve. The strains (1.0 × 10^9^ CFU/ml) were added to the fermentation medium of PNL, mixed well, and then incubated in an incubator at the controlled temperature (30°C) and humidity (relative humidity of 80%) for 144 h. The fermentation products were centrifuged at 4,000 rpm at 4°C for 15 min, freeze-dried at −50°C and 7–10 MPa, and then stored at −80°C until use. The suitable strains were screened to FPNL according to the previous research (Supplementary Table 1; Supplementary Figure 1). Finally, *S. cerevisiae* (CICC 31393) and *B. subtilis* (CICC 22459) were co-inoculated to ferment PNL in this research.

### Total Saponin in PNL and FPNL

The total saponins content of PNL and FPNL were assayed by the vanillin and glacial acetic acid method (19). 1.5 mg ginsenoside Re standard was weighed and added to a 6 ml methanol solution to dissolve entirely. The absorbance was measured at a wavelength of 560 nm, and the standard curve of *P. notoginseng s*aponin was drawn. The content of *P. notoginseng* saponins in ample solutions was determined according to the standard curve drawing method.

### Total Polyphenols in PNL and FPNL

The total phenolic content of PNL and FPNL was assessed using the Folin–Ciocalteu reagent (FCR) method (20). The sample (100 μl) was added to a solution containing FCR (100 μl), sodium carbonate (100 μl; 20% w/v), and methanol (100 μl). The sample–reagent mixture was allowed to react in the dark for 20 min, and then it was centrifuged at 13,000 rpm for 3 min. The absorbance was monitored at 760 nm using gallic acid as a standard, while methanol was used as a blank. The results were calculated as μg of gallic acid equivalent per gram of dry weight.

### Total Flavonoids in PNL and FPNL

The total flavonoids content of PNL and FPNL were assayed by AlCl_3_ colorimetry (21), which is based on the reaction of aluminum ions with flavonoid molecules under primary conditions. A total of 1.5 ml of the extract was added to 450 μl of 5.3% NaNO_2_, 900 μl of 10% AlCl_3_-H_2_O, and 4 ml of 1 M NaOH. The mixture was stirred and allowed to rest for 5 min before each addition. The absorbance was measured at 510 nm.

### Antioxidant Activities in PNL and FPNL

Changes of antioxidant capacity in PNL and FPNL under external environmental stimulation were determined as described by Rakmai et al. (22) with a slight modification. The oxidative stimulation of PNL and FPNL was simulated by UV irradiation for a week. The retention of antioxidant activity of PNL and FPNL under external environmental stimulation were then measured daily from the supernatant after UV irradiation. The antioxidant activity of FPNL and PNL was determined by using a DPPH and ABTS radical scavenging assay (23). 2,2-Diphenyl-1-picrylhydrazyl powder was dissolved in 100 ml methanol to prepare the 0.05 mM DPPH solution for the method. The prepared solution was stored at 4°C. 0.1 ml of sample solution was mixed with 3.9 ml DPPH solution and incubated for 30 min in the dark. 2,2-Diphenyl-1-picrylhydrazyl solution (3.9 ml) and 0.1 ml of 70% methanol aqueous solution were used as negative controls. Then, the absorbance was measured at 517 nm. The scavenging activity (SC) of the samples was expressed using the following formula:


$$\begin{array}{l} \text{The DPPH sactivity }\left(100\%\right)=\left[{\text{A}}_{0}-\left({\text{A}}_{1}-{\text{A}}_{2}\right)/{\text{A}}_{0}\right]\;\times \;100\% \end{array}$$

where A_0_, A_1_, and A_2_ represent the absorbance of the blank control, sample, and negative control, respectively.

The ABTS radicals scavenging was reacting ABTS and potassium persulfate (23). The mixture was incubated in the dark at room temperature. 0.2 ml FPNL and PNL were mixed with ABTS radical cation solution, then incubated for 15 min in the dark. The absorbance was measured at 734 nm. The ABTS activity of the samples was expressed using the following formula:


$$\begin{array}{l} \text{The ABTS activity }\left(100\text{\%}\right)=\left[{\text{A}}_{0}-\left({\text{A}}_{1}-{\text{A}}_{2}\right)/{\text{A}}_{0}\right]\;\times \;100\% \end{array}$$

where A_0_, A_1_, and A_2_ represent the absorbance of the blank control, sample, and negative control, respectively.

### Analysis of Saponins by High Performance Liquid Chromatography

The contents of different saponins in PNL and FPNL were analyzed by the high performance liquid chromatograph (HPLC) method described in a previous study (24, 25). High performance liquid chromatograph-grade methanol and acetonitrile were purchased from Merck (Darmstadt, Germany). Deionized water was purified by a Milli-Q purification system (Millipore, Bedford, MA, USA). Four standard substances (ginsenoside R1, Rg1, Rb1, and Rb3) were purchased from the National Institute for the Control of Pharmaceutical and Biological Products (Beijing, PR China) according to the National Standards of the People's Republic of China (GBT19086-2008).

The sample was dissolved in 70% methanol (v/v) and filtered using Millipore Amicon Ultra UFC900396 (Millipore, Bedford, MA, USA). The centrifugal liquid was filtered by 0.22 μm (Millipore, Bedford, MA, USA) prior to HPLC analysis.

The quantitative analysis of saponins was performed on an Agilent 1260 series HPLC (Santa Clara, CA, USA) equipped with an Agilent Zorbax SB-C18 column (250 × 4.6 mm, 5 μm). The gradient elution system consisted of water (A) and acetonitrile (B). Separation was achieved using the following gradient: 0–6 min, 20–30% B; 6–14 min, 30–40% B; 14–20 min, 40–30% B; and 20–30 min, 20% B. The flow rate was 1.0 ml/min, and the injection volume was 10 μl. The UV detection wavelength was carried out at 203 nm. The column temperature was maintained at 36°C. The contents for ginsenosides in samples were calculated by their respective peak areas (25).

### Metabolomic Analysis by LC-MS/MS

FPNL and PNL (20 mg) were put into EP tube, 1,000 μl solution (acetonitrile:methanol:water = 2:2:1, with the isotopically labeled internal standard mixture) was added. After 30 s mixing, the samples were homogenized at 35 Hz for 4 min and sonicated for 5 min on ice. The homogenization and sonication cycle was repeated 3 times. Then, the samples were incubated for 1 h at −40°C and centrifuged at 12,000 rpm for 15 min at 4°C. The resulting supernatant was transferred to a fresh glass vial for LC/MS analysis. High performance liquid chromatograph separation was carried out using a 1290 Infinity series UHPLC System (Agilent Technologies) with a UPLC BEH Amide column (2.1 × 100 mm, 1.7 μm, Waters). The mobile phase consisted of 25 mmol/L ammonium acetate and 25 mmol/L ammonia hydroxide in water (pH = 9.75) (A) and acetonitrile (B). The analysis was carried out with an elution gradient as follows: 0–0.5 min, 95% B; 0.5–7.0 min, 95–65% B; 7.0–8.0 min, 65–40% B; 8.0–9.0 min, 40% B; 9.0–9.1 min, 40–95% B; 9.1–12.0 min, 95% B. The column temperature was 25°C. The autosampler temperature was 4°C, and the injection volume was 3 μL. A QE mass spectrometer was used for its ability to acquire MS/MS spectra in information-dependent acquisition (IDA) mode in the control of the acquisition software (Xcalibur 4.0.27, Thermo). In this mode, the acquisition software continuously evaluates the full scan MS spectrum. The ESI source conditions were set as follows: sheath gas flow rate of 45 Arb, aux gas flow rate of 15 Arb, capillary temperature of 400°C, full MS resolution of 70,000, MS/MS resolution of 17,500, collision energy of 10/30/60 eV in mode.

### Cell Lines and Culture

Cardiomyocyte H9c2 cell lines were obtained from Kunming Cell Bank of the Typical Culture Preservation Committee, Chinese Academy of Sciences. Cells were cultured in DMEM (HyClone, CA, United States). All cells were cultured in a humidified atmosphere with 5% CO_2_ at 37°C. All media were supplemented with 10% FBS and 1% penicillin-streptomycin mixture.

### MTT Test

H9c2 cells in the logarithmic phase were seeded in 96-well plates. The cell concentration was adjusted to 10,000 cells per well and inoculated with 200 μl (26). Cells were cultured for 24 h and then divided into seven groups: the control group (normal medium and the corresponding volume of DMSO), model group (10 μmol/L ISO), FPNL group (10, 20, 40, 80, 160 μg/ml), and PNL group (10, 20, 40, 80, 160 μg/ml). All groups were incubated for 48 h, then the cells were incubated with 10 μmol/L ISO for 1 h. 5 μg/ml MTT solution was added to each well and continuously incubated for 4 h. Media were replaced with DMSO, and the optical densities (ODs) were measured at 490 nm wavelength using a microplate reader. The inhibition rate was calculated according to the following equation:


$$\begin{array}{l} \text{The inhibition rate of tumor cell proliferation }=\\ \;1-\left(\text{OD drug treatment group}/\text{OD cell control group}\right)\times \;100\% \end{array}$$

### *In-vivo* Animal Swimming Model

To determine the effects of PNL and FPNL on mice *in-vivo*, 4-week-old male C57/BL6 mice (weight 20 g ± 2 g) were fed in an SPF environment (27). All of the animal experiments were performed under protocols approved by the Institutional Animal Ethical Committee of Yunnan Agricultural University (YNAU-201911017). All mice were then randomly divided into five groups (six mice per group): the control group (distilled water), low-dose group (50 mg/kg FPNL), medium-dose group (100 mg/kg FPNL), high-dose group (200 mg/kg FPNL), and not fermentation group (200 mg/kg PNL). The drug was administered by gavage every day. Each group was gavaged continuously for 30 days. Body weights and total food intake of mice were measured every 4 days.

### Weight-Loading Swimming Test

After treatment with FPNL and PNL, all mice rested for 30 min and were then subjected to a weight-loading swimming test (28). Mice were placed in a swimming pool, strapped with 5% of their own body, and swam every week in a swimming tank with a water temperature of 25 ± 1°C and a water depth of 30 cm. The time from the start of swimming to the fatigue of the mice was recorded.

### Serum Indices

After swimming with loaded, mice were killed to collect blood and organs. Blood samples were collected through a heparinization tube for eyeball blood collection and centrifuged at 3,500 rpm for 30 min, and all organs (heart, liver, lungs, spleen, kidney) were frozen in liquid nitrogen (27). The serum contents of LG, LD, LDH, MDA, and blood urea nitrogen (BUN) were investigated by ELISA kits according to the kit instructions. All samples were stored at −80°C.

### Western Blot Analysis

Cells were treated with a water extract of PNL or FPNL for 48 h, and then washed twice with cold PBS. Total cell protein was extracted by RIPA buffer (Beyotime, Shanghai, China), and protein concentrations were assessed by the BCA assay kit (Beyotime, Shanghai, China) (29). Target protein samples were separated by 10% SDS-PAGE and transferred by electroblotting to polyvinylidene difluoride (PVDF) membranes. After blocking with 5% skim milk for 2 h, proteins were incubated with primary antibodies against Bax, Bcl-2, and β-actin overnight at 4°C. The PVDF membranes were washed three times with Tris HCl Tween (TBST) for nearly 8 min each time and then incubated with goat anti-rabbit HRP or goat anti-mouse HRP (1:10,000, Abcam, MA, United States) for 1 h. The protein signal was detected using ECL Western blotting substrate and analyzed by *ImageJ*.

### Statistical Analysis

All data were analyzed using GraphPad Prism 7.0. Values are expressed as the mean ± standard error of the mean (SEM). The results comparisons were performed using one-way analysis of variance (ANOVA). ^*^p < 0.05, ^**^p < 0.01, ^***^p < 0.001, and ^****^p < 0.0001; ^#^*p* < 0.05, ^*##*^*p* < 0.01, and ^*###*^*p* < 0.001 were considered to indicate statistically significant differences. Single factors texts were used to determine the significant differences between means (*P* < 0.05) using SPSS version 17.0 (Chicago, USA). Three replicates (*n* = 3) for *in-vitro* texts, Six replicates (*n* = 6) for *in-vivo* texts.

Principal component analysis (PCA) pattern recognition was performed with SIMCA software (V15.02, Sartorius Stedim Data Analytics AB, Umea, Sweden). Metabolites with VIP values >1.0 were considered differential metabolites for potential discrimination of samples in the OPLS-DA models. We used Student's *t*-test to analyze these models, which is commonly used for metabolomics statistical analysis. *P* < 0.05 was considered statistically significant.

## Results

### Contents of Total Polyphenols, Flavonoids, and Saponins in PNL and FPNL

**Figure 1:**
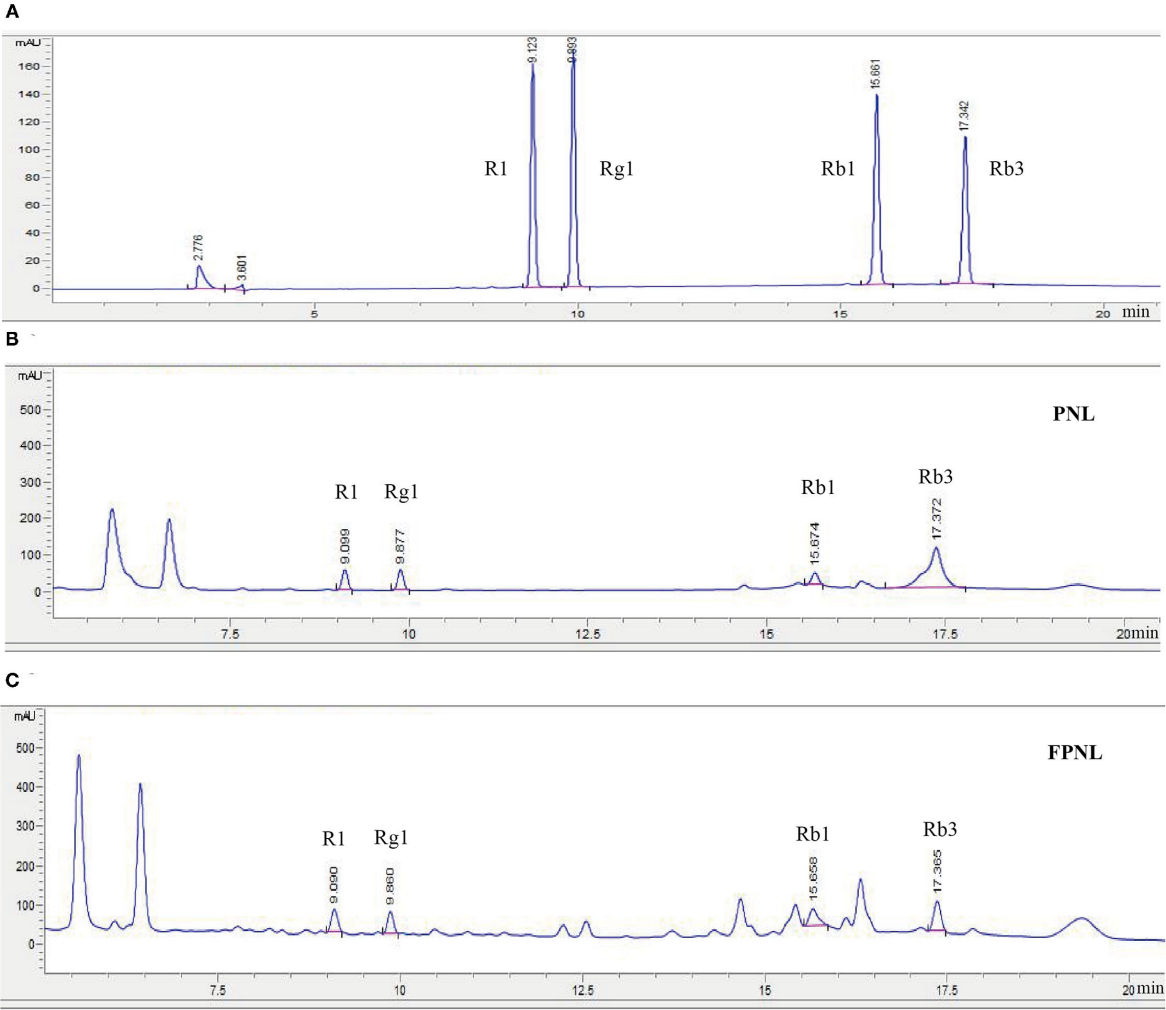
HPLC analysis of different saponins content in PNL and FPNL. **(A)** A stander mixture of four saponins. **(B)** The four saponins in PNL. **(C)** The four saponins in FPNL.

Our previous research found that the highest content of total saponins was obtained from the co-fermentation of PNL by *S. cerevisiae* CICC 31393 and *B. subtilis* CICC 22459 (the ratio of the two microorganisms was 1:1) (Supplementary Table 1; Supplementary Figure 1). Therefore, *S. cerevisiae* CICC 31393 and *B. subtilis* CICC 22459 were used to co-fermented PNL for subsequent studies. The results showed that the total polyphenols, flavonoids, and saponins in FPNL were 1.6, 2.1, and 1.2 times higher than PNL (Table 1). High-performance liquid chromatography analysis showed that after fermentation, the composition of saponins was changed. In particular, compared with the PNL, the PPT type of R1 and Rg1 increased in FPNL, while protopanaxadiol types of Rb1 and Rb3 decreased slightly, indicating that biological transformation of saponins in PNL occurred after fermentation (Figure 1; Supplementary Tables 2, 3).

**Table 1:** Contents of total polyphenols, flavonoids, and saponins in PNL and FPNL.

**Name**	**Total polyphenol content**	**flavonoid content**	**total saponins content**
	**(mg GAE/g)**	**(mg/g)**	**(mg/g)**
PNL	38.26 ± 3.57	25 ± 4.87	161.584 ± 3.13
FPNL	63.75 ± 4.16	54.72 ± 2.85	192.861 ± 2.82

### Antioxidant Stability in PNL and FPNL

**Figure 2:**
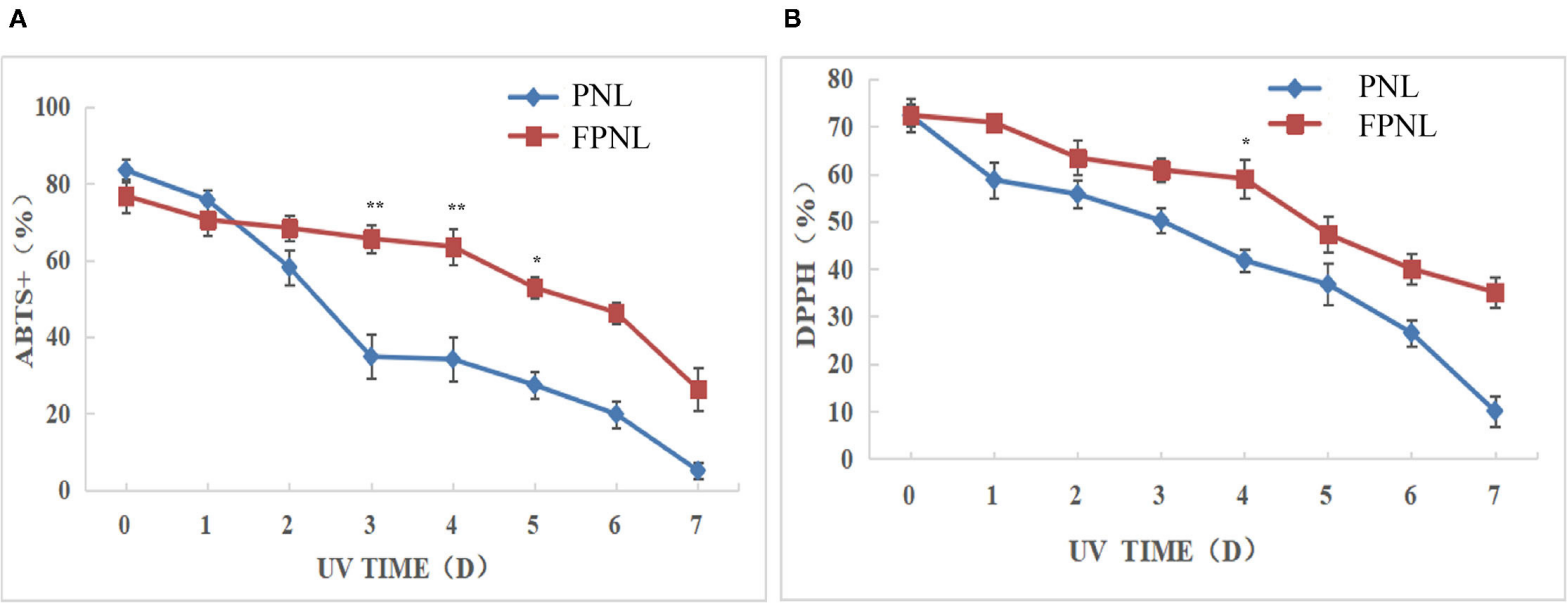
Changes of ABTS and DPPH free radical scavenging rates of PNL and FPNL. **(A)** Changes in ABTS rates of PNL and FPNL. **(B)** Changes in DPPH rates of PNL and FPNL. ^*^P < 0.05, ^**^P < 0.01 vs. PNL group.

Most active substances do not work well due to the influence of water solubility and stability. Therefore, a stability test could be used to compare the stability of active substances produced by fermentation. The oxidative stimulation of PNL and FPNL was performed by UV irradiation. The antioxidant stability of PNL and FPNL was determined by DPPH and ABTS radical scavenging for different days (0–7 days). The results showed that although both DPPH radical scavenging ability and ABTS radical scavenging ability of PNL and FPNL were decreased after UV treatment in time-dependent. The ABTS scavenging ability of PNL decreased rapidly to 5.11%. While, the ABTS scavenging ability of FPNL was relatively stable in the first 4 days and then decreased to 26.34% on the last day (Figure 2A). Similarly, the DPPH radical scavenging ability of PNL was significantly decreased from 72.31 to 10.09%. However, FPNL decreased relatively slowly and still kept at 35.1% on the last day (Figure 2B). In addition, the antioxidant activities of FPNL were also better than PNL between these 7 days. It indicated that FPNL might show significantly antioxidant stability than PNL. These results showed that the active substances in FPNL could be more fully released, and the stability of the active substances could be enhanced to avoid the loss of active substances by the external environment, such as UV, to some extent.

### Change of the Metabolites of PNL After Co-fermented

**Figure 3:**
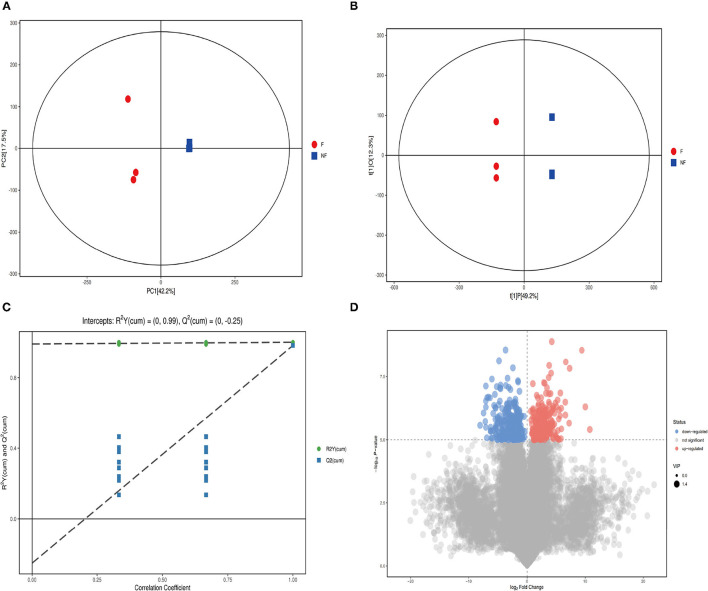
Identification of discriminating biomarkers of fermented *P. notoginseng* leaves metabolomics analysis. **(A)** The OPLS-DA score plot in ion mode. **(B)** The PCA score plot in ion mode. **(C)** The H volcanic figure plot in ion mode. **(D)** The validation plot for the OPLS-DA model is built for negative, the slope of R2 is greater than 0, and the intercept of Q2 on the Y-axis is less than 0.05, indicating a valid model, the scattered shape and color of Figure. Three represent disparate studied groups.

To further to explore the differences in metabolites between PNL and FPNL, LC-MS/MS was used in this study, with the PNL group as a control. As shown in Figure 3, all samples are within the 95% confidence interval. Principal component analysis (Figure 3A) and orthogonal partial least squares discriminant analysis (OPLS-DA) plot scores and volcano plot showed that samples of FPNL and PNL were in different locations, indicating that both models were valid (Figures 3B–D).

**Figure 4:**
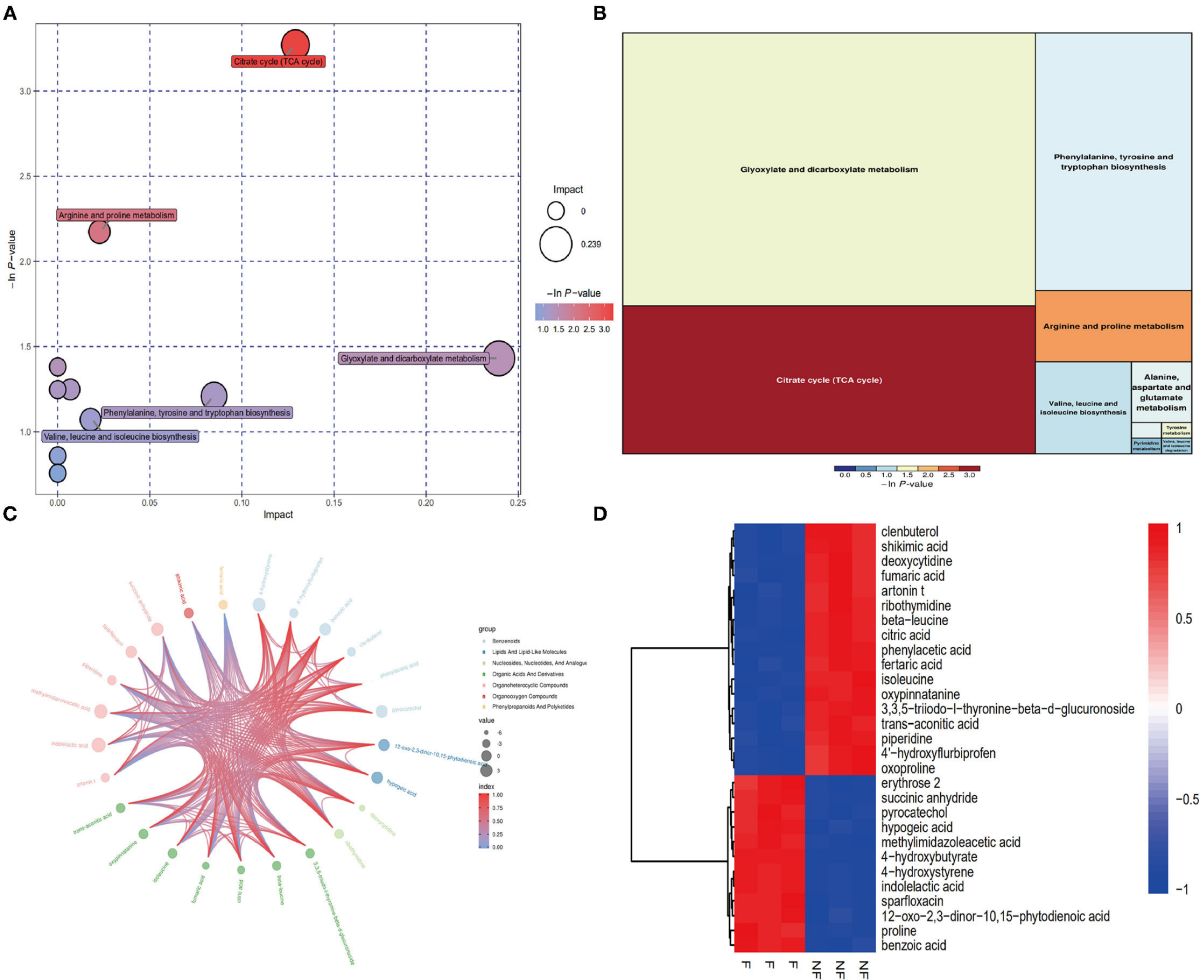
Improvement of different metabolites of PNL and FPNL. **(A)** Bubble plot displays the increasing impact of the enrichment analysis of metabolic pathways, with key bubbles reflecting that the metabolic pathways were significantly altered by fermentation. **(B)** Heatmap clearly shows the relationship in potential biomarkers after fermentation. **(C)** Chord diagram of metabolic differences. **(D)** Metabolites differential enrichment map.

Next, we obtained a comprehensive metabolites view of PNL after fermentation, then mapped these different metabolites into their biochemical pathways (Figures 4A,B). The results of metabolic enrichment and pathway analyses based on the KEGG database and MetaboAnalyst showed significantly changed in the pathways of the citrate cycle (TCA), glyoxylate and dicarboxylate metabolism, arginine, and proline metabolism (Figure 4A; Supplement Figure 2). Moreover, two kinds of essential AAs (Isoleucine, Leucine) and six kinds of unessential AAs (Tyrosine, Valine Leucine Alanine, Glutamate Proline) pathways were enriched in FPNL (Figure 4B). As shown in the string map and heat map (Figures 4C,D), some active metabolites were rich in FPNL, too. A total of 31 different metabolites were selected based on variable importance in the projection (VIP) values >1 and *p*-values <0.05. The significantly different metabolites with known nutritional functions identified in the FPNL are shown in Table 2. These metabolites mainly include phenolic acids, amino acids, and pyrimidines. In this study, the contents of organic phenolic acids such as pyrocatechol, citric acid, benzoic acid, shikimic acid, and the contents of AAs such as Isoleucine, Leucine, Proline in FPNL were significantly increased.

**Table 2:** The variations in *P. notoginseng* leaves metabolites after fermented.

**Name**	**VIP**	***P*** **-value**	**Fold**	**Log**
4′Hydroxyflurbiprofen	1.4236	0.000322	0.2550	−1.9712
Beta-leucine	1.4254	0.000140	0.2909	−1.7812
4-Hydroxystyrene	1.4244	0.00031	14.1641	3.8241
Deoxycytidine	1.4163	0.00027	0.0351	−4.8291
Piperidine	1.4214	0.00030	0.3596	−1.4752
Pyrocatechol	1.4226	0.000319	5.1849	2.3742
Artonin t	1.4218	0.000362	0.2299	−2.1204
3,3,5-Triiodothyronine-beta-glucuronoside	1.4214	0.00210	0.3509	−1.5107
Clenbuterol	1.4236	0.0002367	0.20421	−2.29187
Methylimidazoleacetic acid	1.3927	0.0001182	29.4480	4.8809
Proline	1.4248	0.0002815	3.46094	1.79116
Oxoproline	1.4214	0.0002703	0.16272	−2.61946
Citric acid	1.4212	0.0001197	0.118788	−3.07352
Ribothymidine	1.4240	0.0002998	0.32737	−1.61098
Hypogeic acid	1.4232	0.0002173	3.26973	1.70917
Fumaric acid	1.4107	0.0002565	0.067328	−3.89263
Isoleucine	1.1699	0.0002199	0.48346	−1.04850
Indolelactic acid	1.4203	0.0000845	53.38945	5.73841
Phenylacetic acid	1.4241	0.0001078	0.008551	−6.86956
Benzoic acid	1.4235	0.0002099	3.448989	1.786173
Oxypinnatanine	1.4225	0.0001560	0.457686	−1.275697
Shikimic acid	1.4244	0.0002616	0.583155	−0.778048
4-Hydroxybutyrate	1.2262	0.0000176	682.8043	9.41532
Sparfloxacin	1.4233	0.0002161	2.82629	1.49891
Erythrose 2	1.4237	0.0003457	4.366523	2.12648
Trans-aconitic acid	1.4185	0.0002928	0.359388	−1.476384
Succinic anhydride	1.4244	0.0001543	7.521064	2.910931
Fertaric acid	1.4232	0.00018291	0.272257	−1.876595
12-Oxo-2,3-dinor 10,15-phytodienoic acid	1.4191	0.00029548	2.504321	1.32443
Analyte	1.4247	0.00012273	0.09360	−3.417341

### FPNL Alleviate Myocardial Cell Failure

**Figure 5:**
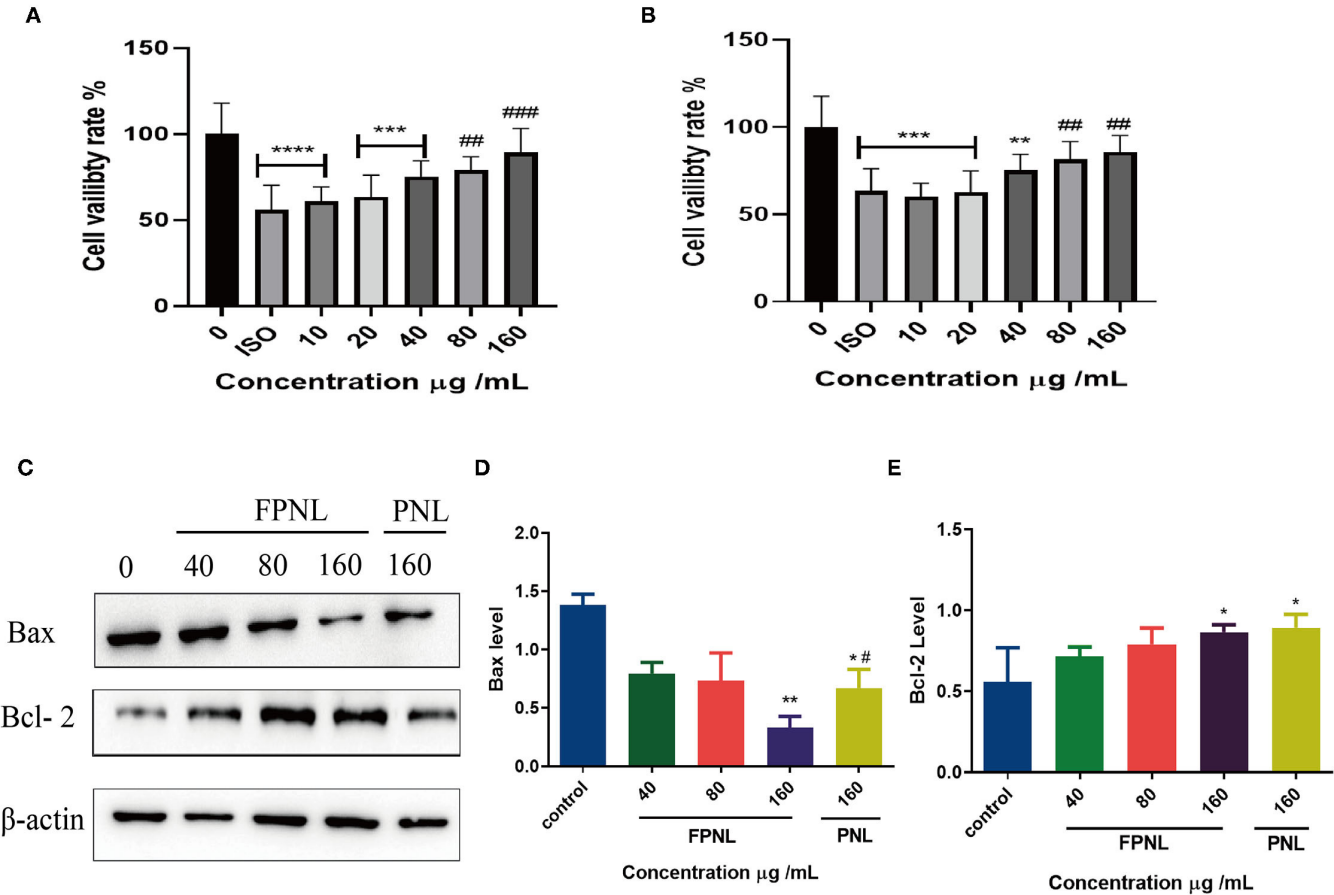
The effect of PNL and FPNL on ISO-induced H9c2 cell apoptosis. **(A)** Alleviated of the proliferation of H9c2 cells by different concentrations of FPNL. **(B)** Alleviated of the proliferation of H9c2 cells by different concentrations of PNL. **(C–E)** Expression of apoptotic proteins (Bax, Bcl-2) in HCT116 cells. Data are expressed as mean ± SEM from three independent experiments (*n* = 3). ^*^P < 0.05, ^**^P < 0.01, ^***^P < 0.001, ^****^P < 0.0001 vs. control. ^**#**^*P* < 0.05, ^**##**^*P* < 0.01, ^**###**^*P* < 0.001 vs. FPNL (160 μg/ml) group.

To determine the antifatigue effect of FPNL, H9c2 cells were treated with different concentrations of aqueous extracts of PNL and FPNL for 48 h, and then treated with ISO for 1 h. MTT assay showed that, compared with the control group, the ISO-treatment group showed lower cell viability (Figures 5A,B). The viability of cells in the FPNL group (Figure 5A) and PNL group was increased significantly and showed less toxicity on H9c2 cells (Figure 5B). Moreover, compared with the PNL group, the FPNL group had a significant antifatigue effect on H9c2 cells. Based on these results, 40, 80, and 160 μg/ml concentrations for FPNL and PNL were selected for subsequent experiments.

### The Effect of FPNL on Myocardial Cell Apoptosis

To detecte whether FPNL affects apoptosis in H9c2 cells, the expression of the apoptosis-related proteins Bax and Bcl-2 was examined by Western blot. The results showed that the expression of pro-apoptotic protein Bax was decreased (Figures 5C,D) when the concentration was 160 μg/ml, the expression of Bax in the FPNL group was better than that in the PNL group, and the expression of anti-apoptotic protein Bcl-2 was significantly increased (Figures 5C,E). It was suggested that the antifatigue effect of FPNL on the ISO-induced cells might be driven by the intrinsic apoptotic pathway.

### The Effect of FPNL on Weight-Loading Swimming Mice

**Figure 6:**
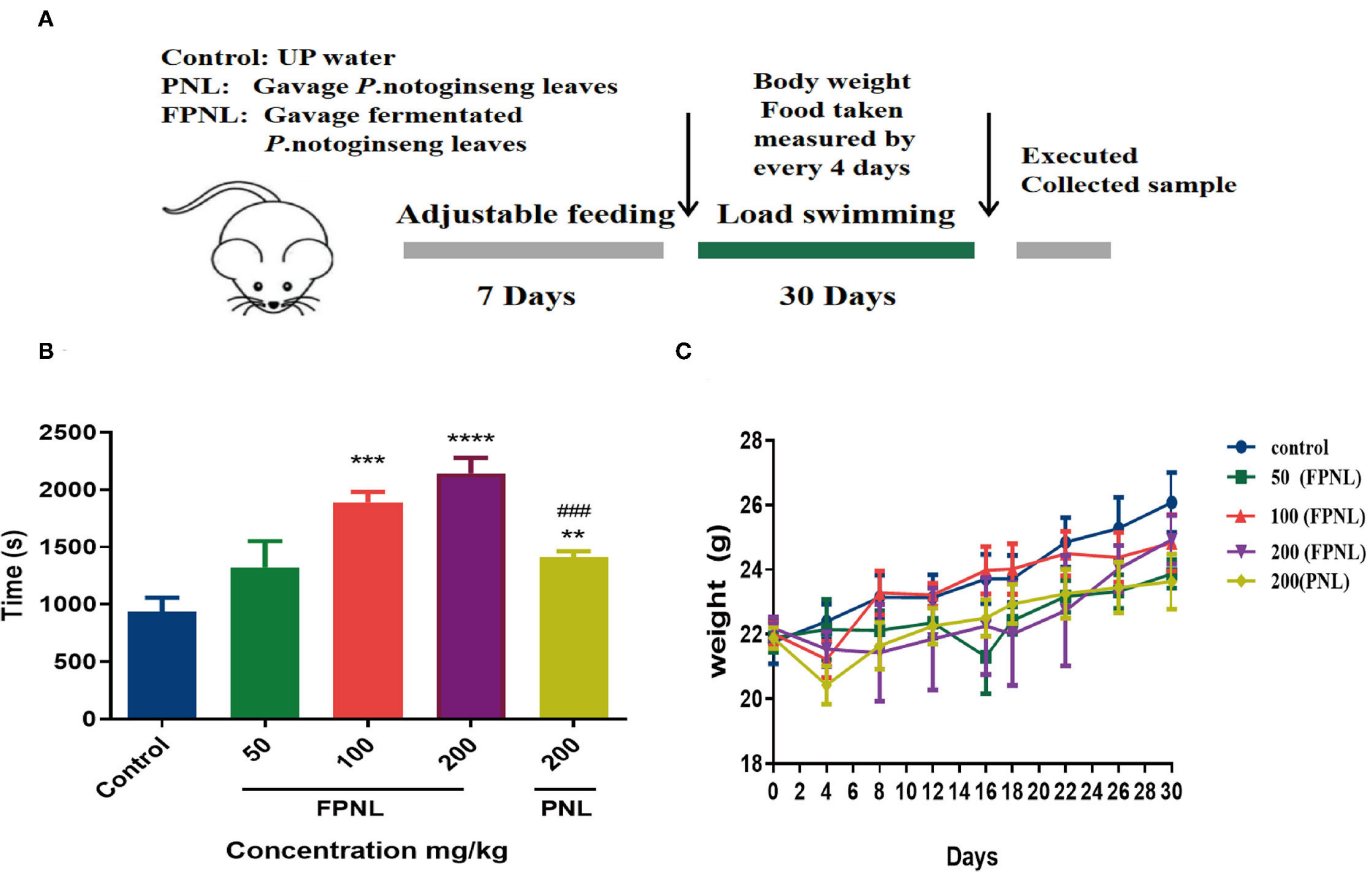
Fermented *P. notoginseng* leaves alleviates fatigues in swimming mice. **(A)** Loading swimming experimental design. **(B)** Swimming time with weight loading after gavage PNL and FPNL. **(C)** Body weight, measured every 4 days. Data are expressed as mean ± SEM from six independent parallel experiments (*n* = 6). ^**^P < 0.01, ^***^P < 0.001, ^****^P < 0.0001 vs. control. ^**###**^*P* < 0.001 vs. FPNL (200 mg/kg) group.

The animal swimming experiment is one of the important indices to evaluate the antifatigue effect of healthy food (30). Therefore, we evaluated the swimming ability of mice after gavaging FPNL and PNL (Figure 6A). The results showed that the swimming time of mice with 100 and 200 mg/kg FPNL and 200 mg/kg PNL groups was significantly longer than the control group (*P* < 0.05). Moreover, the FPNL groups showed significantly longer swimming time than the PNL groups in a dose-dependent manner. The swimming time increased by 1.96 times in FPNL group (200 mg/kg) compared with PNL group (200 mg/kg) (Figure 6B) (*P* < 0.05). These results indicated that FPNL showed a significantly relieving fatigue effect *in-vivo*. As shown in Figure 6C, after 30 days of gavage, the average body weights of mice in the control group, FPNL (50, 100, and 200 mg/kg) groups, and PNL (200 mg/kg) groups were 22.94, 23.51, 22.73, and 22.16 g, respectively. The organ weight of the mice did not significantly change (Table 3). These results suggested that FPNL may improve the antifatigue function and exhibit no toxicity in the mice in the dose range used in this study.

**Table 3:** Organ weight of mice.

**Orange weight**	**Control**	**FPNL**		**PNL**	
**(g)**		**50 (mg/kg)**	**100 (mg/kg)**	**200 (mg/kg)**	**200 (mg/kg)**
Heart	0.23 ± 0.20	0.22 ± 0.13	0.21 ± 0.13	0.25 ± 0.12	0.24 ± 0.22
Liver	1.62 ± 0.1	1.65 ± 0.08	1.64 ± 0.14	1.67 ± 0.13	1.63 ± 0.17
Lungs	0.31 ± 0.15	0.33 ± 0.11	0.35 ± 0.16	0.38 ± 0.15	0.35 ± 0.18
Spleen	0.16 ± 0.2	0.19 ± 0.07	0.15 ± 0.11	0.17 ± 0.17	0.18 ± 0.15
Kidney	0.58 ± 0.13	0.60 ± 0.20	0.61 ± 0.12	0.63 ± 0.21	0.56 ± 0.14

### The Effect of FPNL on LG, MDA, and BUN Levels

**Figure 7:**
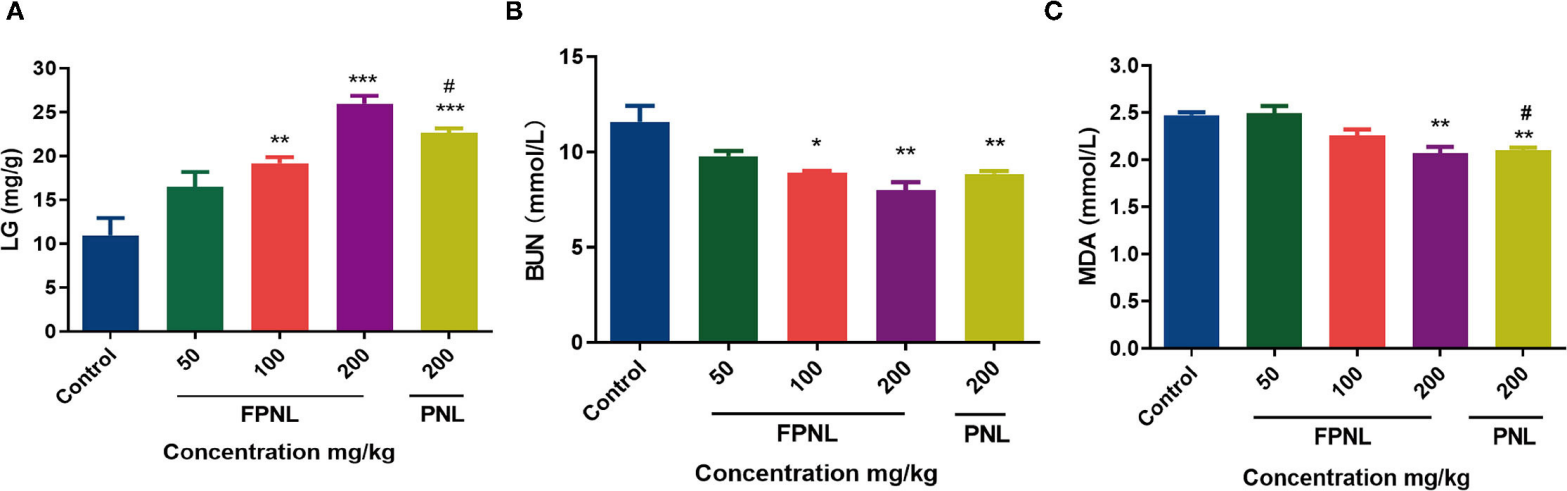
Effect of fermented *P. notoginseng* leaves and *P. notoginseng* leaves on LG BUN, MDA level in serum. **(A)** The LG level in mice serum after swimming. **(B)** The BUN level in mice serum after swimming. **(C)** The MDA level in mice serum after swimming. Data are expressed as mean ± SEM from six independent experiments (*n* = 6). ^*^P < 0.05, ^**^P < 0.01, ^***^P < 0.001 vs. control. ^**#**^*P* < 0.05 vs. FPNL (200 mg/kg) group.

Liver glycogen content is one of the indicators of physical fatigue. After weight-loading swimming, the LG of mice was consumed, which makes fatigue occur. As shown in Figure 7A, the LG levels of mice in the FPNL and PNL were significantly higher than those in the control group (*P* < 0.05). Furthermore, the LG levels of FPNL were significantly higher than PNL at the same dose (200 mg/kg) (*P* < 0.05).

The effects of FPNL and PNL on BUN and MDA levels in mice after swimming are shown in Figures 7B,C. The BUN level of the mice in the FPNL groups was significantly lower than the control group (*P* < 0.05). Additionally, compared to the PNL groups, the BUN level in the mice of the FPNL group at 200 mg/kg significantly decreased (Figure 7B). Malondialdehyde levels in the FPNL groups were also reduced significantly compared with the control group (*P* < 0.05) (Figure 7C), although there was no significant difference at concentrations of 50 and 100 mg/kg.

### The Effect of FPNL on LD and LDH Levels in Mice

**Figure 8:**
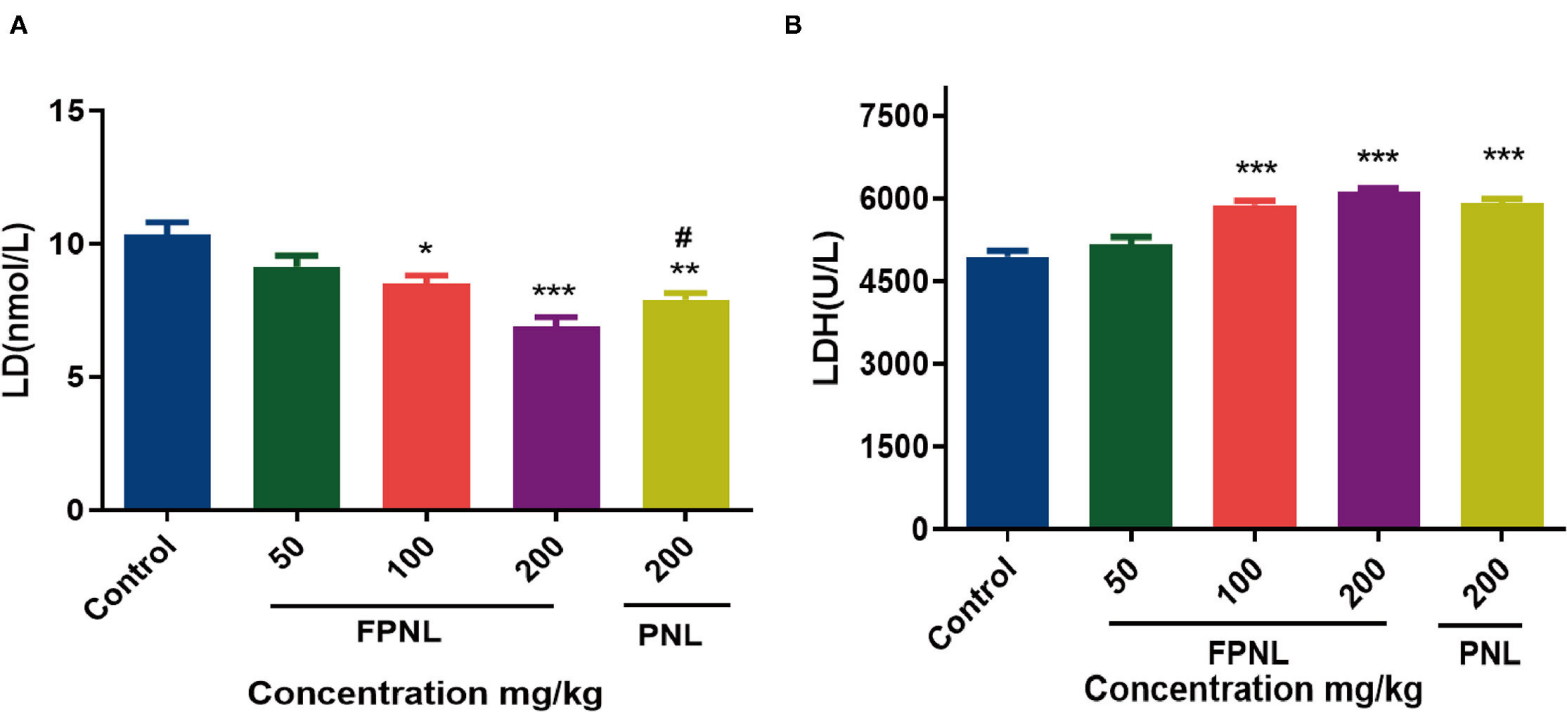
Effect of fermented *P. notoginseng* leaves and *P. notoginseng* leaves on LD, LDH level in serum. **(A)** The LD level in mice serum after swimming. **(B)** The LDH level in mice serum after swimming. Data are expressed as mean ± SEM from six independent experiments (*n* = 6). ^*^P < 0.05, ^**^P < 0.01, ^***^P < 0.001 vs. control. ^**#**^*P* < 0.05 vs. FPNL (200 mg/kg) group.

Lactic acid was produced after fatigue exercise. Compared with the control group, the LD level in the FPNL and PNL groups significantly decreased (*P* < 0.05) (Figure 8A). Moreover, the LD level of the FPNL groups was dramatically lower than the PNL at the same dose (200 mg/kg). The results illuminated that FPNL effectively impeded the accumulation of LD with a dose-dependent decrease and played a positive role in delaying fatigue.

Lactate dehydrogenase is a glycolytic enzyme that oxidizes LD into pyruvate. Therefore, LDH can be used as an indicator of fatigue level. The results showed that compared with the control group, the LDH content in mice with high- and medium-dose FPNL and PNL groups was significantly increased (Figure 8B). Furthermore, compared with the PNL, the LDH level of FPNL was significantly increased at the same dose (200 mg/kg). These results indicated that the FPNL could significantly increase the activity of LDH, accelerate the removal of LD, and improve the antifatigue ability of mice.

## Discussion

*Panax notoginseng* leaves as a new resource food approved by the China Ministry of Health, widely used in food processing. It showed the different effects of antioxidant, antidiabetes, and immunity (31–33). In this study, we reported for the first time that the co-fermentation of PNL by fungi and bacteria was enriched with saponins, phenolic acids, and amino acids, and found that FPNL may have strong antifatigue and antioxidant capacity. Moreover, FPNL could reduce ISO-induced damage to H9c2 myocardial cells by regulating the apoptotic pathway, prolong the weight-loading swimming time of mice, and exhibit antifatigue effects *in intro* and *in-vivo*.

Microbial fermentation is a common way to improve the nutrition and quality of foods (34, 35). Fermentation by a single strain can lead to a large accumulation of microbial metabolites, which in turn feedback to inhibit the synthesis of related enzymes and affect fermentation effectiveness. Although mixed fermentation was coordinated in growth and metabolism, some microorganisms such as yeasts can use metabolites to increase growth and eliminate feedback inhibition (36). In this study, single and mixed strains were used to ferment PNL. The results showed that the total saponin content of PNL was highest with co-fermented by *S. cerevisiae* and *B. subtilis* (Supplementary Table 1; Supplementary Figure 1). *S. cerevisiae* and *B. subtilis* are widely used as safety microorganisms in fermented foods. However, the long growth cycle of *S. cerevisiae* affects the supply and synthesis efficiency of food-grade functional factors, while *B. subtilis* has the advantages of a short growth period and fast growth rate. This implies that *B. subtilis* and *S. cerevisiae* have a synergistic and symbiotic effect during co-fermentation (37). *Bacillus subtilis* can secrete biological enzymes such as cellulase, amylase, and protease, which can decompose cellulose and other carbohydrates in plants. The oligosaccharides and monosaccharides produced after decomposition can be utilized by *S. cerevisiae*, thus eliminating the influence of the glucose inhibition effect and improving the fermentation efficiency. Meanwhile, co-fermentation can produce abundant metabolites that not only degrade macromolecular proteins into small peptides or amino acids, but also produce proteins that are more conducive to digestion and absorption (38). Therefore, in this study, *S. cerevisiae* and *B. subtilis* were used to co-ferment PNL to assess the antifatigue *in-vitro* and *in-vivo*.

Microbial fermentation not only secretes more enzymes to destroy plant cells and release active components, but it is also accompanied by the modification and transformation of plant active components. Some plant components can stimulate microorganisms to produce secondary metabolites. Through fermentation, various biomolecules such as phenolic compounds, saponins, flavor substances, organic acids, and pigments can be produced or enriched (34). As reported by Xia et al. (39), the antioxidant activities of soymilk are significantly enhanced due to the increase in phenolics and aglucone flavone after microbial fermentation. In the present study, organic phenolic acids in PNL, such as pyrocatechol, citric acid, benzoic acid, shikimic acid, were significantly increased by co-fermentation (Table 2), thus increasing the types and contents of phenolic acids in the FPNL. Studies have shown that these different phenolic acids have antifatigue effects (31, 32). In particular, pyrocatechol (40, 41) and citric acid (42) could significantly alleviate exercise fatigue by improving the antioxidant capacity.

Also, LC-MS/MS results showed that microorganisms also biotransformed the active substances in PNL through TCA cycle, glucose-alanine cycle and amino acid cycle (Figure 4). Specifically, two essential AAs (Isoleucine, Leucine) and six kinds of unessential AAs (Tyrosine, Valine Leucine Alanine, Glutamate Proline) were enriched during all the fermentation processes (Table 2). Previous studies have also shown that fermentation leads to changes in the amino acid content of *P. notoginseng* (43, 44). Thus, these changes in components of FPNL may improve its antifatigue effect.

In addition, microbial fermentation has shown significant advantages in the biosynthesis of triterpenoid saponins. It was shown that the level of saponins C-K increased six-fold in PNL after fermentation by *Lactobacillus brevis* M3 (45). Kim et al. (46) FPNL with *L. plantarum* M1 and found that the total ginsenosides content increased to 176.8 mg/g after 4 days. Our results also demonstrated that co-fermentation increased total saponins content in PNL (Table 1). The total saponins in PNL also showed potent antifatigue activity that prolonged the exhaustive swimming time of mice (27). In addition, some saponins of FPNL were altered in this study. The levles of R1 and Rg1 were increased, while the levels of Rb1 and Rb3 were decreased (Figures 1B,C; Supplementary Table 2). Some studies reported that Rb1 and Rg1 had significant antifatigue effects on hypoxia mice (28). Interestingly, R1 and Rg1 were 20 (S)—protopanaxatriol types (PPT), Rb1 and Rb3 were 20 (S)—protopanaxadiol types (PPD). Rb1 could be converted to R1 and Rg1, and Rg1 could be converted to Rb1 by biotransformation pathway after fermentation (47). Studies have shown that either PPT or PPD could improve the antifatigue ability of mice and prolong exercise time by modulating the levels of corticosterone, LD, and creatinine. However, the protective effect of the PPT was stronger than that of PPD (48). Our results also showed that R1 and Rg1 increased in FPNL (Figure 1; Supplementary Table 2).

Furthermore, plant components can also activate gene expression by altering microbial secondary metabolism pathways to produce new secondary metabolites. Wang et al. (25) found that *P. notoginseng* by steaming significantly altered the composition of saponins. After treatment, new saponins as 20S/R-Rh1, Rk3, Rh4, 20S/R-Rg3, Rk1, and Rg5 appeared. These new saponins are promising to improve hyperlipidemia. The Rg3 content of *P. notoginseng* roots was changed after fermentation (49). Similarly, changes were found in this study by HPLC result. Compared with the PNL group, some new peak patterns appeared around the four saponins in the FPNL group, and these new peaks may be the saponins produced by biotransformation after co-fermentation (Figure 1B). Therefore, we can further determine the structures of these saponins by NMR spectrometry and spectrum technology, and explore the molecular mechanisms between saponins and antifatigue properties by molecular docking techniques in the future.

When oxidative factors rise, or the efficacy of antioxidant defenses falls in cells, oxidative stress increases, and oxidative stress negatively affects muscle contractility. Fatigue has been demonstrated to be closely related to free radicals (50). Oxidative stress in muscles severely interferes with muscle activity during exercise, reducing exercise performance or increasing fatigue (51). Our results showed that FPNL showed better oxidation stability than PNL (Figure 2). Isoproterenol is a catecholamine that can enhance cardiac contractility and cardiovascular systolic blood pressure by oxidative damage (52). Therefore, ISO is often used to induce myocardial fibrosis and heart failure models (53). *Panax notoginseng* leaves could protect against LPS-induced astrocyte damage by activating the Nrf2 pathway and antioxidant system to inhibit ROS, and showed no toxicity of cells (54). Jin et al. indicated that PNL (0–80 μg/ml) could protect H9c2 cells against hypoxia-reoxygenation-induced cell damage, accompanied by increased cell viability, reduced cell apoptosis, and downregulation of LDH and MDA (55). Our study also found that different concentrations of FPNL significantly repaired ISO-induced damage in the H9_C_2 myocardial cell model. Compared with the PNL group, the FPNL group showed a significant antifatigue effect on H9c2 cells. Moreover, the cell survival rate was above 80%, indicating that FPNL exhibited low toxicity to H9c2 cells (Figures 5A,B). Furthermore, the expression of the pro-apoptotic protein Bax in the cells tended to decrease. In contrast, the expression of the anti-apoptotic protein Bcl-2 was significantly increased in the FPNL group (Figures 5C,D). These results suggested that FPNL alleviated ISO-induced damage possibly driven by reducing oxidative damage and regulating the intrinsic apoptotic pathway.

In addition, the improvement of exercise-related endurance is the most visual indicator for the antifatigue effect of functional foods. The weight-loading swimming test has been widely used to evaluate exercise ability and validate the antifatigue effects of natural functional foods (56–58). Zhou et al. (28) found that PNL at dose of 0.42, 1.11, and 11.53 g/kg showed a significant effect on improving swimming time, and there was no apparent toxic effect on mice. Other studies have also suggested that PNL with gavage doses of 20–80 mg/kg showed antifatigue activity, prolonging the exhaustive swimming time in mice (27). Moreover, Yang et al. (59) gavaged mice at 1 or 2 g/kg/day for 30 days, and found that none of the mice died. In this study, FPNL showed a significant antifatigue effect on weight-loading swimming mice at the dose of 50–200 mg/kg (Figure 6A). In addition, each group had normal living habits, and there were no significant changes in organs or bodyweight of mice (Figure 6C; Table 3). It indicated that FPNL showed no toxicity on the mice and displayed harmless effects (Figure 6).

The primary energy used by organisms for exercise comes from decomposition fats and carbohydrates. Glycogen is the primary energy resource for the body during exercise (56). Glycogen is excess glucose in the body that accumulates and is stored in the liver. When the body needs energy, glycogen decomposes into glucose to produce energy (60). The increase in LG consumption has been associated with increased fatigue (27). In this study, 200 mg/kg of FPNL significantly improved LG reserve and slowed down the duration of fatigue in mice (Figure 7A). Blood urea nitrogen is the final ammonia product of human protein metabolism. Our study showed that the BUN levels in FPNL groups were significantly lower than those in PNL groups (Figure 7B). Several studies have shown that strenuous exercise could accelerate the production of free radicals and further induce oxidative damage. Malondialdehyde level is a typical indicator of the degree of lipid peroxidation (5). In this study, FPNL significantly reduced the MDA levels compared with the control and PNL groups, suggesting that FPNL could retarding MDA production. The overaccumulation of ammonia produced by amino acid metabolism could reduce endurance and lead to fatigue (Figure 7C). Lactic acid and LDH are the primary metabolites of glycolysis during exercise, which are the key indicators of physical fatigue (61). Excess LD could decrease the pH value and weaken muscle contraction strength. LDH could consume LD to relieve fatigue (62). In this study, we found that FPNL significantly reduced LD levels (Figure 8A). In contrast, FPNL increased LDH levels to clearance the LD in mice after exercise (Figure 8B). These results showed that FPNL shows a strong antifatigue effect by regulating oxidative factors *in-vivo*.

## Conclusion

In this study, it was found that the contents of saponins, phenolic acids, flavonoids, and other active substances in PNL were significantly increased and showed good antioxidant stability capacity after co-fermentation by *S. cerevisiae* and *B. subtilis*. Moreover, FPNL could repair ISO-induced damage in H9c2 myocardial cells by reducing the expression of Bax and improving the expression of Bcl-2 to avoid cell apoptosis *in-vitro*. *In-vivo* experiments showed that FPNL could significantly improved the swimming-loading time and enhanced metabolic ability in mice by reducing the levels of BUN, MDA, and LD, and improving the levels of LG and LDH. This study strongly indicated that PNL co-fermented with *S. cerevisiae* and *B. subtilis* could improve exercise-related energy storage and alleviate fatigue.

## Data Availability Statement

The original contributions presented in the study are included in the article/Supplementary Materials, further inquiries can be directed to the corresponding author/s.

## Ethics Statement

The animal study was reviewed and approved by Institutional Animal Ethical Committee of Yunnan Agricultural University (YNAU-201911017).

## Author Contributions

JS and YT supervised the study. YT, L-FL, LT, and MY designed experiments. MY, LT, C-CZ, Z-LW, Z-JY, WZ, and Y-LW performed experiments. MY wrote the manuscript. YT, L-FL, and LT reviewed the results and the manuscript. All authors contributed to the article and approved the submitted version.

## Funding

This work was supported by Major Project of Science and Technology Department of Yunnan Province (202002AA100005 and 202102AE090027), YEFICRC Project of Yunnan Provincial Key Programs (2019ZG009), and Yunnan Province Young and Middle-aged Academic and Technical Leaders Reserve Talents Project (2018HB040).

## Conflict of Interest

The authors declare that the research was conducted in the absence of any commercial or financial relationships that could be construed as a potential conflict of interest.

## Publisher's Note

All claims expressed in this article are solely those of the authors and do not necessarily represent those of their affiliated organizations, or those of the publisher, the editors and the reviewers. Any product that may be evaluated in this article, or claim that may be made by its manufacturer, is not guaranteed or endorsed by the publisher.

## Abbreviations

BUN: blood urea nitrogen
LD: lactic acid
LDH: lactate dehydrogenase
LG: liver glycogen
MDA: malondialdehyde
Bcl-2: B-cell lymphoma 2
ABTS, 2: 2-azino-bis-3-ethylbenzthiazoline-6-sulphonic salt
DPPH, 2: 2-diphenyl-1-picrylhydrazyl
HPLC: high-performance liquid chromatography
DMEM: Dulbecco's modified Eagle's medium
DMSO: dimethyl sulfoxide
PBS: phosphate buffered saline
PPT: protopanaxatriol
PPD: protopanaxadiol
PNL: *Panax notoginseng* leaves
FPNL: fermented *P. notoginseng* leaves.
