# Supplementary material for: Antifatigue Effect of Panax Notoginseng Leaves Fermented With Microorganisms: In-vitro and In-vivo Evaluation
Source: Front Nutr. 2022 Feb 22;9:824525. doi: 10.3389/fnut.2022.824525 (PMC8904179; doi:10.3389/fnut.2022.824525)
Supplement: Supplementary file 1 [file Data_Sheet_1.docx]

**SUPPLEMENT INFORMATION**

**Antifatigue effect of *Panax notoginseng* leaves fermented with microorganisms: *in vitro* and *in vivo* evaluation**

**Min Yang^1, 2†^, Liang Tao^1, 2, 3†^, Cun-Chao Zhao^2, 3^, Zi-Lin Wang^1, 2^, Zhi-Jin Yu^1, 3^, Wen Zhou^1^, Yan-Long Wen^1, 2^, Ling-Fei Li^1, 2^*, Yang Tian^1, 2, 3^* and Jun Sheng^2, 4^***

^1^ College of Food Science and Technology, Yunnan Agricultural University, Kunming, China

^2^ Engineering Research Center of Development and Utilization of Food and Drug Homologous Resources, Ministry of Education, Yunnan Agricultural University, Kunming, China

^3^ National Research and Development Professional Center for Moringa Processing Technology, Yunnan Agricultural University, Kunming, China

^4^ Key Laboratory of Pu-er Tea Science, Ministry of Education, Yunnan Agricultural University, Kunming, China

*** Correspondence:**

**Dr. Ling-Fei Li,** E-mail: lingfeili@163.com;

**Dr. Yang Tian,** E-mail: [tianyang1208@163.com](mailto:tianyang1208@163.com);

**Dr. Jun Sheng,** E-mail: [shengj@ynau.edu.cn](mailto:shengj@ynau.edu.cn)

† These authors contributed equally to this work.

**Table S1. Changes of total saponins fermented by different strains**

| **Different strains** | **Total saponins (mg/g)** |
| --- | --- |
| *Saccharomyces cerevisiae*  (CICC 31393) | 167.861±12.07 |
| *L. plantarum* (CICC 194165) | 125.222±5.03 |
| *RhizopusOryzae* (CICC 41441) | 153.625±3.41 |
| *Pichia kluyveri* (CICC 32845) | 121.958±5.13 |
| *Lactobacillus acidophilus*  (CICC 20710) | 125.986±1.46 |
| *Bacillus subtilis* (CICC 22459) | 173.834±16.64 |
| *L. reuteri* (CICC 6226) | 125.570±2.13 |
| *S. cerevisiae* (GIM2.43) | 143.694±3.24 |
| **Different proportions**  CICC 31393：CICC 41441（1:1） | 176.889±2.34 |
| CICC 31393：CICC 22459（1:1） | 192.861±2.82 |
| CICC 41441：CICC 22459（1:1） | 181.125±2.92 |


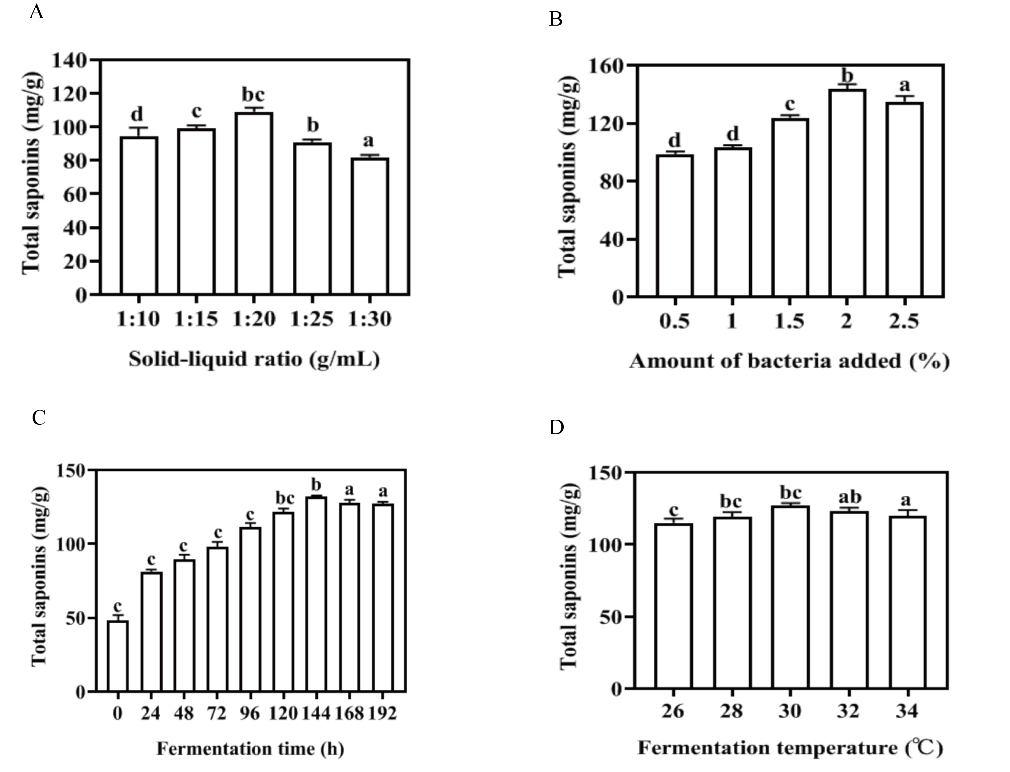


**Fig. S1. Single factor test of microbial fermentation of *P.* notoginseng leaves. (A)** Influence of solid-liquid ratio on total saponins of FPNL. **(B)** Influence of bacteria added on total saponins of FPNL. **(C)** Influence of fermentation time on total saponins of FPNL. **(D)** Influence of fermentation temperature on total saponins of FPNL. Mean values with different small letters were significantly at different level *P* <0.05.

**These above data were not published**

**Table S2. saponins contents of PNL by HPLC analysis**

| Name  (PNL) | Retention  time(min) | Peak area  (mAU*s) | Concentration  (mg/g) |
| --- | --- | --- | --- |
| R1 | 9.099 | 66.2196 | 11.27799188 |
| Rg1 | 9.877 | 73.5379 | 12.12376011 |
| Rb1 | 15.674 | 97.10281 | 17.18528735 |
| Rb3 | 17.372 | 536.20685 | 112.0888965 |

**Table S3. saponins contents of FPNL by HPLC analysis**

| Name  (FPNL) | Retention  time(min) | Peak area  (mAU*s) | Concentration  (mg/g) |
| --- | --- | --- | --- |
| R1 | 9.090 | 89.88052 | 15.34875783 |
| Rg1 | 9.860 | 95.25162 | 15.66619682 |
| Rb1 | 15.658 | 235.32139 | 40.91135505 |
| Rb3 | 17.365 | 365.48035 | 76.51642914 |


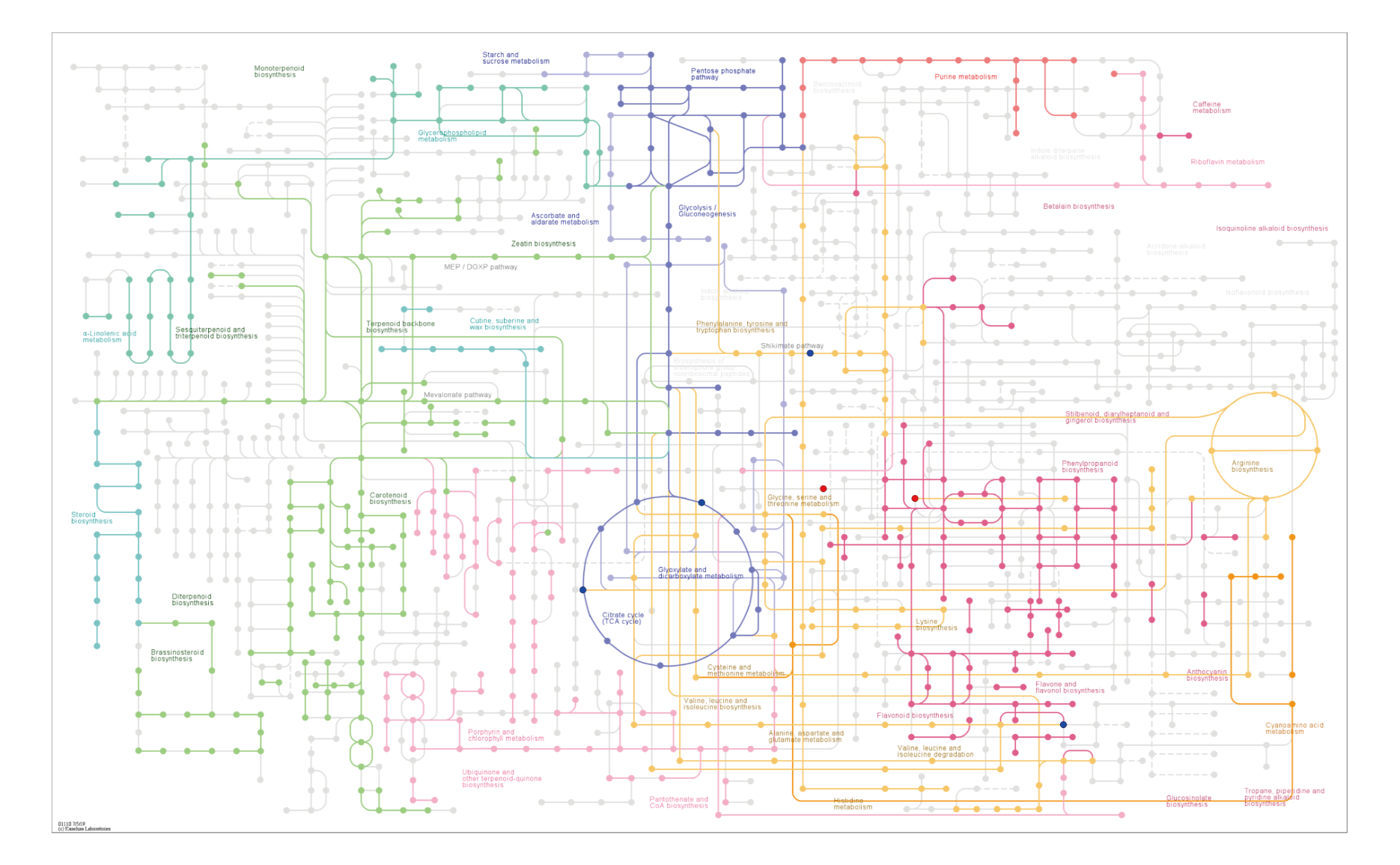


**FigS2. KEGG pathway cross-linked enrichment network of fermentation *P.* notoginseng leaves.**
